# A Comparison between BMI, Waist Circumference, and Waist-To-Height Ratio for Identifying Cardio-Metabolic Risk in Children and Adolescents

**DOI:** 10.1371/journal.pone.0149351

**Published:** 2016-02-22

**Authors:** Luís B. Sardinha, Diana A. Santos, Analiza M. Silva, Anders Grøntved, Lars B. Andersen, Ulf Ekelund

**Affiliations:** 1 Exercise and Health Laboratory, CIPER, Faculdade de Motricidade Humana, Universidade de Lisboa, Cruz-Quebrada, Portugal; 2 Centre of Research in Childhood Health, Institute of Sports Science and Clinical Biomechanics, University of Southern Denmark, Odense, Denmark; 3 MRC Epidemiology Unit, Institute of Metabolic Science, Addenbrooke’s Hospital Hills Road, University of Cambridge, Cambridge, United Kingdom; 4 Department of Sport Medicine, Norwegian School of Sport Sciences, Oslo, Norway; University, ITALY

## Abstract

**Background:**

There is controversial evidence on the associations between anthropometric measures with clustering of cardiovascular disease risk factors in pediatric ages. We aimed to examine the associations between body mass index (BMI), waist circumference (WC), and waist-to-height ratio (WHtR) with clustered cardiometabolic risk factors and to determine whether these anthropometric variables can be used to discriminate individuals with increased cardiometabolic risk (increased clustered triglycerides, HDL-cholesterol, systolic and diastolic blood pressure, and HOMA-IR).

**Methods:**

The study sample of 4255 (2191 girls and 2064 boys) participants (8–17 years) was derived from pooled cross-sectional data comprising five studies. Outcomes included a continuous cardiometabolic risk factor z-score [corresponding to the sum of z-scores for triglycerides, HDL-cholesterol, systolic and diastolic blood pressure (mean arterial pressure), and HOMA-IR] and children with ≥1.0 SD in this score were defined as being at risk for clustering cardiometabolic risk factors.. Exposure variables were BMI, WC, WHtR. Statistics included mixed-effect regression and ROC analysis.

**Results:**

All anthropometric variables were associated with clustered risk and the magnitudes of associations were similar for BMI, WC, and WHtR. Models including anthropometric variables were similar in discriminating children and adolescents at increased risk with areas under the ROC curve between 0.70 and 0.74. The sensitivity (boys: 80.5–86.4%; girls: 76.6–82.3%) was markedly higher than specificity (boys: 51.85–59.4%; girls: 60.8%).

**Conclusions:**

The magnitude of associations for BMI, WC, and WHtR are similar in relation to clustered cardiometabolic risk factors, and perform better at higher levels of BMI. However, the precision of these anthropometric variables to classify increased risk is low.

## Introduction

Childhood overweight and obesity are predictive of adulthood type 2 diabetes mellitus, hypertension, and dyslipidemia [1,2]. Additionally, children and adolescents with a high proportion of visceral fat and a relatively low proportion of subcutaneous fat may suffer from severe metabolic complications [3].

Screening for metabolic complications early in life is considered clinically important [4]. However, it is unclear whether practitioners should focus on assessing body mass index (BMI) or waist circumference as alternative non-invasive measures for obesity-related cardiometabolic risk. The International Diabetes Federation [5] stated abdominal obesity as the “sine qua non” when defining pediatric metabolic syndrome (MetS). The World Health Organization (WHO) [6] emphasizes the assessment of BMI in younger ages, providing BMI-for-age and sex percentiles. Similarly, the Centers for Disease Control and Prevention [7] has presented BMI-for-age growth charts developed in a multi-year process, with recommendations to define childhood overweight and obesity based on a BMI-for-age and sex above the 85^th^ and 95^th^ percentiles. A high BMI-for-age has been investigated to be associated with the risk for biochemical abnormalities and adult obesity [8–11]. Additionally, the use of a waist-to-height ratio (WHtR) has also been advocated to take into account differences in age and sex [9,12,13].

It has been suggested that it is unlikely that WC and BMI provide a valid quantification of visceral fat depot and that WC is a better marker for total body adiposity than it is for visceral fat. Further, both WC and BMI appear to perform equally well for estimating children and adolesents total and abdominal visceral fat [14]. These observations may have implications when diagnosing obesity-related cardiometabolic complications in pediatric ages.

The literature is diverging about the magnitude of associations between different anthropometric measures and cardiometabolic risk in pediatric ages. Some studies indicated stronger associations between measures of abdominal adiposity (i.e. WHtR vs BMI) with several cardiovascular risk factors [13,15,16]. Kahn et al. [16] verified that WHtR provided a better estimate than BMI for a number of risk factors, including LDL cholesterol (4.1% vs 6.6%), triglycerides (10.5% vs 15.0); also, Hara et al. [15] observed that comparing to BMI and WC, WHtR provided a better estimate of a cardiovascular risk score (WHtR: 36% vs BMI:28% and WC: 26%). Contrarily, other authors have suggested no difference between BMI, WC and WHtR in relation to cardiometabolic risk [9,17,18]. There is however little evidence on the different associations between these different anthropometric measures with clustering of cardiovascular disease risk factors in a large sample of children and adolescents living in different cultural settings.

The hypothesis of this study was that the magnitude of the associations between BMI, WC and WHtR with composite cardiometabolic risk score were similar and that these anthropometric variables can be used to discriminate between individuals with and without increased risk in a large heterogeneous sample of children and adolescents aged 8 to 17 years old.

## Materials and Methods

### Participants

Cross-sectional data from 4255 (2191 girls and 2064 boys) children and adolescents (8–17 years) were obtained from five studies: European Youth Heart Study [19] [Denmark (n = 1327), Estonia (n = 629), and Portugal (n = 1171], and National Health and Nutrition Examination Survey [20] (NHANES 03/04 (n = 550), and NHANES 05/06 (n = 578)]. These studies were performed between 1998 and 2009. Participants were included if they had valid data for all outcome variables.

### Ethics Statement

All participants and parents or guardians were informed about the possible risks of the investigation before giving written informed consent to participate. Formal data sharing agreements were established and all partners consulted their individual research board to confirm that sufficient ethical approval had been attained for contributing data. For the EYHS all procedures were approved by the ethics committee from the University of Southern Denmark, the institutional review board from the Technical University of Lisbon, and from the ethics committee from the University of Tartu. For the NHANES data the National Center for Health Statistics (NCHS) ethics review board approved the survey protocols. The study was conducted in accordance with the declaration of Helsinki for human studies of the World Medical Association [21].

### Measurements

#### Anthropometry

Height and weight were measured using standardized clinical procedures across studies. Body mass index was calculated and categorized into normal, overweight, or obese according to the WHO standards [6]. Age adjusted BMI z-scores were calculated by regressing BMI on age by sex and standardized residuals from the model represented age adjusted BMI z-score.

In all studies except for the NHANES, waist circumference was measured according to the WHO procedures [22]. In NHANES (n = 1128) waist circumference was assessed according to the National Institutes of Health (NIH) procedure [23] and an equation was applied [24] so that all WC results refer to the WHO procedures.

Age adjusted WC z-scores were calculated by regressing WC on age by sex and standardized residuals from the model represented age adjusted WC z-score.

Waist-to-height ratio (WHtR) was calculated [WC (cm)/height (cm)] and transformed into WHtR z-score. WHtR was additionally categorized into two categories that allow classifying the same proportion of children and adolescents as the BMI categories (overweight/obese).

#### Cardiometabolic markers

Systolic (SBP) and diastolic blood pressure (DBP) were measured while seated, according to established criteria [19,25,26]. SBP and DBP were regressed on height by sex. Residuals from these models represented height adjusted SBP (_adj_SBP) and DBP (_adj_DBP).

Blood samples were collected from the antecubital vein after an overnight fast. Glucose, insulin, triglycerides and HDL- cholesterol (HDL-C) were measured in all participants according to standard clinical procedures [19,25,27].

Homeostasis model assessment of insulin resistance (HOMA-IR) was calculated as [fasting glucose (mmol/L)×fasting insulin (mU/L)]/22.5 as previously described [28].

Triglycerides and HOMA-IR were log transformed before analysis due to skewed distribution.

#### Continuous Cardiometabolic Risk Score

Plasma triglycerides, HDL-C (inverted, iHDL-C), Blood pressure (systolic and diastolic), and HOMA-IR were used to derive a composite cardiometabolic risk score. Each variable was converted into an age adjusted z-score by sex.

Age adjusted risk factors z-scores were calculated by regressing risk factors on age by sex and standardized residuals from the model represented age adjusted risk factors z-score. An age adjusted continuous cardiometabolic risk score (composite z-score) was calculated for each participant as follows:

Composite z-score = z-triglycerides+z-iHDL-C+(z-_adj_SBP+z-_adj_DBP*2)/3+z-HOMA-IR (3)

The cardiometabolic risk score was dichotomized at the cutoff value mean + 1SD, to identify participants with elevated clustered risk, as previously suggested [29].

### Statistical analyses

Analyses were performed with Stata/IC software (vs.13, 2013; Statacorp LP, College Station, TX, USA). Descriptive statistics [mean (95% Confident Intervals)] were calculated for all measurements. Normality was verified using the Q-Q plots and the histograms of each variable. Participants were included in analysis if they had valid data for all outcome variables. The 10^th^, 25^th^, 50^th^, 75^th^, and 90^th^ percentile were calculated for the main outcome variable (composite z-score).

The data was first modeled by using quadratic functions and analyses were conducted by sex (using the age adjusted variables). Mixed effect linear regression models were performed by sex and BMI category and by sex and percentile, to test the association of BMI, WC, and WHtR with the composite z-score for metabolic risk factors and with each individual risk factor, adjusting for the study as a random factor. Linear regression analysis by sex, BMI category, and study were also conducted.

Multivariate logistic regression analysis were conducted to develop a global classification model (by sex and age categories) that allowed the identification of BMI, WC, or WHtR cutoff that correctly identifying children and adolescents with composite z-score ≥1SD. Stepwise backward (likelihood ratio) method was used to retain the variables (age, BMI/WC/WHtR, and BMI^2^/WC^2^/WHtR^2^) that were significant in the final model. The predicted probabilities of each model were used in ROC analyzes to determine each models’ discriminative capability. Sensitivity and specificity were calculated for a range of cutoffs for the exposures BMI, WC, and WHtR). The AUC for the ROC curves and their standard deviation and 95% confident intervals were calculated to determine the overall precision of anthropometric variables in diagnosing true positive participants (composite z-score ≥1SD). The decision for the optimal threshold was the cutoff value with the highest accuracy that maximized the sum of the sensitivity and specificity. Age and sex specific cutoffs for BMI, WC, and WHtR were presented by solving the logistic regression analysis to predict the probability corresponding to the cutoff that maximized the sum of sensitivity and specificity. Significance was set at p<0.05 for all tests.

## Results

A total of 4255 children and adolescents (8–17 years) from five studies were considered for data analysis. Table 1 presents the characteristics of the study participants.

**Table 1:** Participants’ demographic characteristics [mean (95% confident intervals)].

	Boys (n = 2064)	Girls (n = 2191)
Age (years)^a^	12.4 (12.2, 12.5)	12.4 (12.3, 12.5)
Weight (kg)	49.3 (48.4, 50.1)	46.2 (45.5, 46.9)
Height (cm)	154.2 (153.4, 155.0)	150.0 (149.4, 150.5)
BMI (kg/m^2^)	19.9 (19.7, 20.1)	20.0 (19.8, 20.2)
WC (cm)	68.5 (67.9, 69.0)	66.4 (65.9, 66.9)
WHtR	0.44 (0.44, 0.45)	0.44 (0.44, 0.45)
Triglycerides (mmol/L)	0.81 (0.79, 0.83)	0.84 (0.83, 0.86)
HDL-C (mmol/L)	1.46 (1.44, 1.47)	1.48 (1.47, 1.50)
SBP (mmHg)	105.9 (105.4, 106.5)	102.6 (102.2, 103.0)
DBP (mmHg)	58.6 (58.2, 58.9)	60.0 (59.7, 60.3)
Insulin (mU/L)	8.65 (8.36, 8.94)	9.52 (9.26, 9.78)
Glucose (mmol/L)	5.20 (5.18, 5.21)	5.06 (5.04, 5.08)
HOMA-IR	2.04 (1.96, 2.11)	2.17 (2.10, 2.23)
Composite score	0.00 (-0.10, 0.10)	0.00 (-0.10, 0.10)

^a^ Each age was categorized by the midpoint of an age. For example, the group of children with 8 years old included all the children between 7.50 years and 8.49.

Abbreviations: WC, waist circumference; WHtR, waist-to-height ratio; HDL-C high-density lipoprotein cholesterol; SBP, systolic blood pressure; DBP, diastolic blood pressure, HOMA-IR, homeostasis model assessment: insulin resistance.

The race of the participants of the current manuscript was available in 72.9% of the sample (27.1% missing data). Of this 72.9% a total of 69.6% were Caucasian, 13.1% were Hispanic, 13.0% were African-American/European, 0.5% were Asian, and 3.8% other race.

High associations were observed between all anthropometric variables, adjusting for sex and age (BMI with WC: r = 0.91; BMI with WHtR: r = 0.90; and WC with WHtR: r = 0.94).

**Fig 1:**
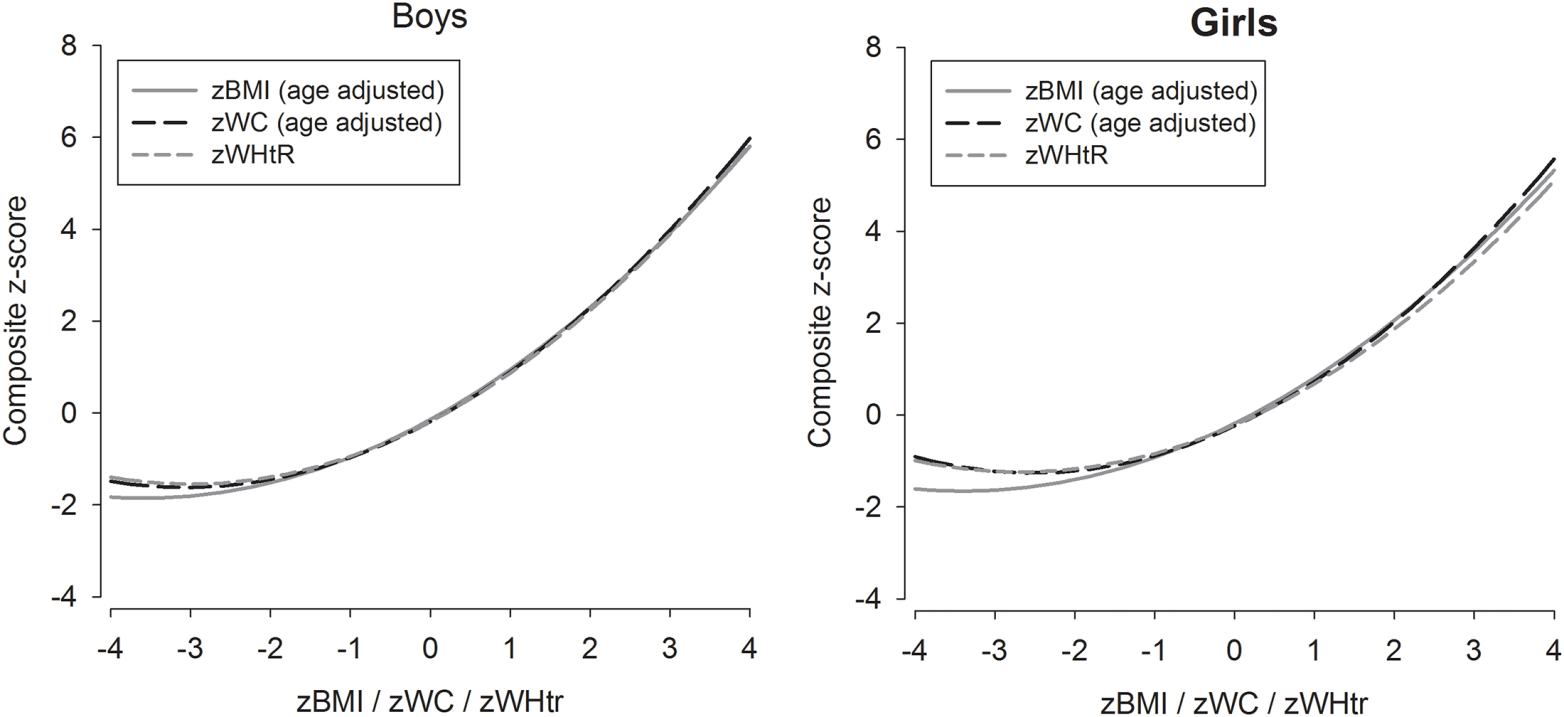
Associations between body mass index, waist circumference, and waist-to-height ratio z-scores with age adjusted clustering of risk factor composite z-score for boys (n = 2064) and girls (n = 2191).

**Fig 2:**
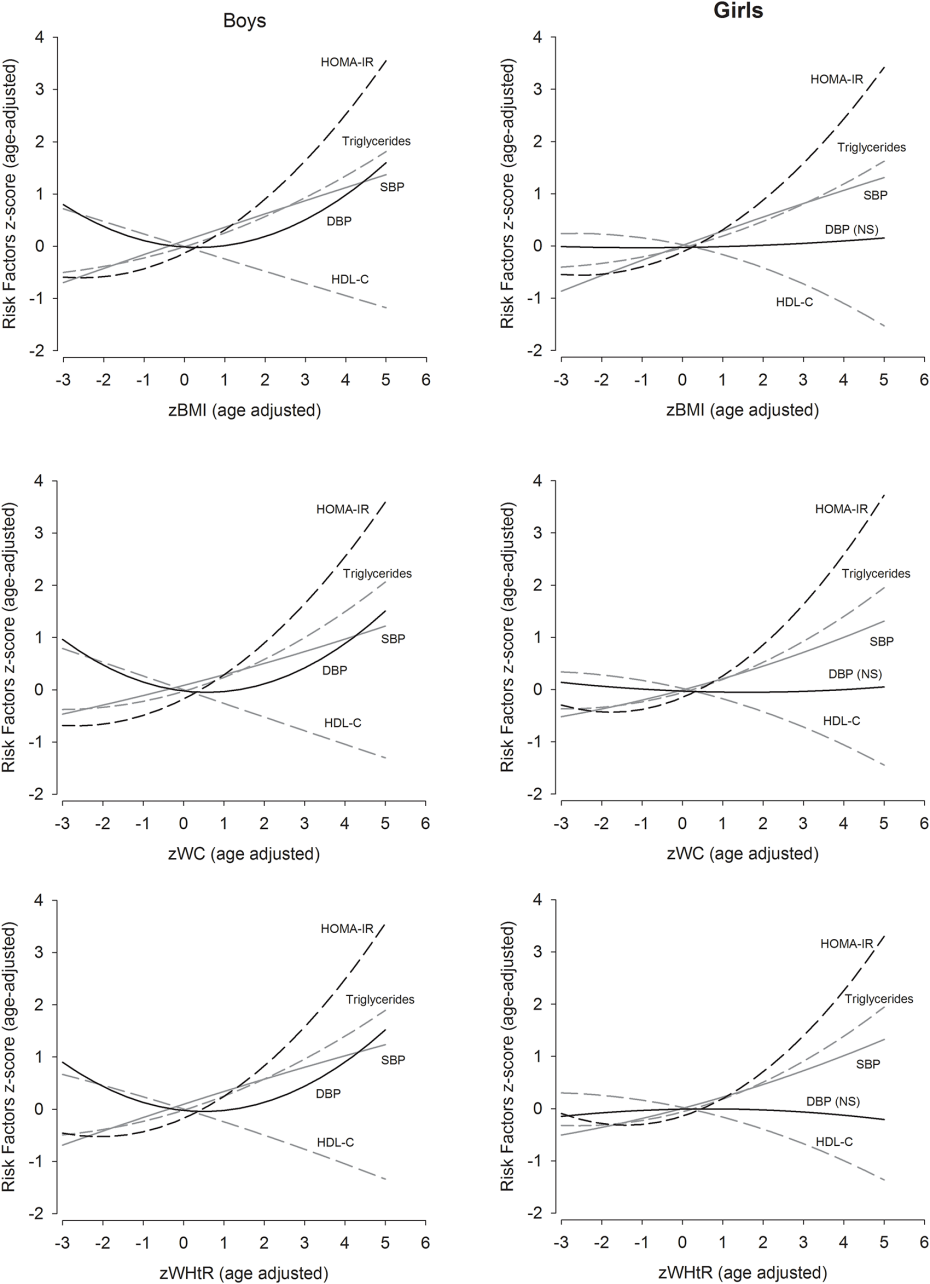
Association between body mass index, waist circumference, and waist-to-height z-scores with age adjusted risk factor measures for boys (n = 2064) and girls (n = 2191). NS: non-significant.

Fig 1 shows the curvilinear associations of BMI, WC, and WHtR with the composite cardiometabolic risk score stratified by sex while Fig 2 illustrates the association of the anthropometric variables with each individual metabolic risk factor, suggesting similar magnitudes of associations for the anthropometric variables for each individual risk factor.

Given the quadratic association between anthropometric variables and the composite cardiometabolic risk score we stratified the sample according to BMI categories (normal weight vs overweight/obese) to explore whether the associations between anthropometrics and the cardiometabolic risk variables differed according to obesity status (Table 2).

**Table 2:** Standardized Coefficients [β (95% confident interval)] for the association between body mass index, waist circumference, and waist-to-height ratio with cardiometabolic variables by sex and body mass index categories ^a^.

	Normal Weight			Overweight / Obese		
	*Body Mass Index*	*Waist Circumference*	*Waist-to-Height Ratio*	*Body Mass Index*	*Waist Circumference*	*Waist-to-Height Ratio*
**Boys**						
Composite z-score	0.13 (0.08, 0.18) ^b^	0.11 (0.06, 0.16) ^b^	0.09 (0.04, 0.14) ^b^	0.47 (0.39, 0.54) ^b^	0.51 (0.44, 0.58) ^b^	0.50 (0.43, 0.57) ^b^
Triglycerides	0.06 (0.01, 0.11) ^b^	0.05 (0.00, 0.11) ^b^	0.07 (0.02, 0.12) ^b^	0.28 0.21, 0.36) ^b^	0.32 (0.25, 0.40) ^b^	0.31 (0.24, 0.39) ^b^
HDL-C	-0.12 (-0.18, -0.07) ^b^	-0.14 (-0.19, -0.08) ^b^	-0.10 (-0.16, -0.05) ^b^	-0.26 (-0.33, -0.18) ^b^	-0.29 (-0.36, -0.22) ^b^	-0.27 (-0.35, -0.20) ^b^
SBP	0.11 (0.07, 0.16) ^b^	0.03 (-0.02, 0.08)	0.09 (0.04, 0.14) ^b^	0.17 (0.09, 0.25) ^b^	0.11 (0.03, 0.19) ^b^	0.17 (0.09, 0.25) ^b^
DBP	-0.07 (-0.12, -0.02) ^b^	-0.12 (-0.16, -0.07) ^b^	-0.10 (-0.15, -0.05) ^b^	0.18 (0.11, 0.26) ^b^	0.16 (0.08, 0.24) ^b^	0.20 (0.12, 0.28) ^b^
HOMA-IR	0.08 (0.03, 0.13) ^b^	0.10 (0.05, 0.15) ^b^	0.04 (-0.01, 0.09) ^b^	0.47 (0.41, 0.54) ^b^	0.52 (0.45, 0.59) ^b^	0.47 (0.40, 0.54) ^b^
**Girls**						
Composite z-score	0.13 (0.08, 0.18) ^b^	0.08 (0.03, 0.13) ^b^	0.05 (0.00, 0.10)	0.41 (0.33, 0.48) ^b^	0.45 (0.37, 0.53) ^b^	0.40 (0.31, 0.48) ^b^
Triglycerides	0.06 (0.01, 0.11) ^b^	0.07 (0.02, 0.12) ^b^	0.06 (0.01, 0.12) ^b^	0.22 (0.14, 0.30) ^b^	0.30 (0.22, 0.39) ^b^	0.28 (0.20, 0.37) ^b^
HDL-C	-0.05 (-0.10, 0.00) ^b^	-0.06 (-0.11, -0.01) ^b^	-0.03 (-0.08, 0.03)	-0.25 (-0.33, -0.17) ^b^	-0.26 (-0.34, -0.17) ^b^	-0.25(-0.34, -0.16) ^b^
SBP	0.13 (0.08, 0.18) ^b^	0.02 (-0.03, 0.07)	0.07 (0.02, 0.12) ^b^	0.18 (0.09, 0.26) ^b^	0.13 (0.04, 0.21) ^b^	0.16 (0.07, 0.25) ^b^
DBP	0.00 (-0.05, 0.04)	-0.07 (-0.12, -0.01) ^b^	-0.01 (-0.06, 0.04)	0.03 (-0.05, 0.11)	0.00 (-0.09, 0.08)	-0.01 (-0.10, 0.08)
HOMA-IR	0.13 (0.08, 0.18) ^b^	0.08 (0.02, 0.13) ^b^	-0.02 (-0.08, 0.03)	0.43 (0.36, 0.51) ^b^	0.47 (0.40, 0.55) ^b^	0.34 (0.26, 0.42) ^b^

^a^ Body mass categories were defined according to the World Health Organization criteria;

^b^ significant association at p< 0.05.

Abbreviations: HDL-C high-density lipoprotein cholesterol; SBP, systolic blood pressure; DBP, diastolic blood pressure, HOMA-IR, homeostasis model assessment: insulin resistance

The associations between anthropometric variables with the composite cardiometabolic score, were considerably stronger in overweight and obese participants compared with their normal weight counterparts (Table 2). For normal weight participants standardized regression coefficients ranged from 0.09 (WHtR) to 0.13 (BMI) in boys and from 0.05 (WHtR, non-significant) to 0.13 (BMI) in girls. For overweight and obese participants the associations were greater in magnitude; coefficients ranging from 0.47 (BMI) to 0.51 (WC) in boys and from 0.40 (WHtR) to 0.45 (WC). Overall, within each sex and BMI category the magnitude of associations between BMI, WC, and WHtR with the composite cardiometabolic risk were similar.

Considering each individual risk factor the weakest associations were observed for DBP. For all other risk factors the associations between BMI, WC, and WHtR with each individual risk factor, a greater magnitude of association was observed in overweight and obese compared to normal weight children and adolescents.

In table 3 are presented the associations between BMI, WC, and WHtR with the composite z-score by study.

**Table 3:** Correlation Coefficients for the association between body mass index, waist circumference, and waist-to-height ratio with composite z-score by sex, body mass index categories and by study^a^.

	Normal Weight			Overweight / Obese		
	*BMI*	*WC*	*WHtR*	*BMI*	*WC*	*WHtR*
**Boys**						
Denmark EYHS	0.17^b^	-0.05^c^	-0.06^c^	0.29^b^	0.14^d^	0.14^d^
Estonia EYHS	0.13	0.18^b^	0.18^b^	0.54^b^^,^^d^	0.62^b^^,^^d^	0.48^b^^,^^d^
Portugal EYHS	0.09	0.17^b^	0.09	0.43^b^^,^^d^	0.47^b^^,^^d^	0.46^b^^,^^d^
NHANES 05/06	0.10	0.21^b^	0.22^b^	0.54^b^^,^^d^	0.63^b^^,^^d^	0.61^b^^,^^d^
NHANES 03/04	0.18^b^	0.30^b^	0.30^b^	0.53^b^^,^^d^	0.59^b^^,^^d^	0.61^b^^,^^d^
**Girls**						
Denmark EYHS	0.17^b^	-0.02^c^	-0.04^c^	0.32^b^	0.14	0.11^c^
Estonia EYHS	0.04	0.12^b^	0.12^b^	0.32^b^	0.47^b^^,^^d^	0.40^b^
Portugal EYHS	0.07	0.02	0.01	0.46^b^^,^^d^	0.44^b^^,^^d^	0.40^b^^,^^d^
NHANES 05/06	0.22^b^	0.27^b^	0.18^b^	0.49^b^^,^^d^	0.55^b^^,^^d^	0.47^b^^,^^d^
NHANES 03/04	0.23^b^	0.28^b^	0.21^b^	0.32^b^	0.44^b^	0.37^b^

^a^Body mass categories were defined according to the World Health Organization criteria;

^b^significant association;

^c^significantly different from BMI;

^d^significantly different from normal weight.

Abbreviations: BMI, body mass index; WC, waist circumference; WHtR, waist-to-height ratio; EYHS, European Youth Heart Study; NHANES, National Health and Nutrition Examination Survey.

Although there were some differences in the magnitude of the associations between studies, with the exception of the Denmark EYHS, no differences were observed in the correlation coefficients for the association between BMI, WC, and WHtR with the composite z-score. Overall, the magnitude of the association was higher in overweight/obese compared to normal weighted participants.

We additionally explored whether the associations differed by composite cardiometabolic score percentiles (table 4),

**Table 4:** Standardized Coefficients [β (95% confident interval)] for the association between body mass index, waist circumference, and waist-to-height ratio with composite z-score by sex and composite z-score percentiles

	*BMI*	*WC*	*WHtR*
**Boys**			
<10^th^	0.06 (-0.08, 0.20)	0.02 (-0.11, 0.16)	0.03 (-0.10, 0.17)
10^th^-25^th^	0.05 (-0.06, 0.16)	0.13 (0.02, 0.24)^a^	0.10 (-0.01, 0.21)
25^th^-50^th^	-0.01 (-0.09, 0.08)	0.01 (-0.08, 0.10)	0.01 (-0.08, 0.10)
50^th^-75^th^	0.06 (-0.03, 0.15)	0.02 (-0.07, 0.11)	0.05 (-0.04, 0.13)
75^th^-90^th^	0.09 (-0.02, 0.20)	0.08 (-0.03, 0.19)	0.08 (-0.03, 0.19)
≥90^th^	0.40 (0.27, 0.53)^a^	0.40 (0.26, 0.53)^a^	0.43 (0.29, 0.57)^a^
**Girls**			
<10^th^	0.17 (0.04, 0.31)^a^	0.17 (0.03, 0.30)^a^	0.14 (0.01, 0.27)^a^
10^th^-25^th^	-0.04 (-0.15, 0.06)	-0.05 (-0.16, 0.06)	-0.03 (-0.14, 0.08)
25^th^-50^th^	0.03 (-0.06, 0.11)	0.04 (-0.05, 0.12)	0.02 (-0.06, 0.11)
50^th^-75^th^	0.04 (-0.04, 0.13)	0.01 (-0.08, 0.10)	0.05(-0.04, 0.14)
75^th^-90^th^	0.08 (-0.03, 0.20)	0.08 (-0.04, 0.20)	0.08 (-0.04, 0.20)
≥90^th^	0.33 (0.21, 0.46)^a^	0.35 (0.23, 0.47)^a^	0.30 (0.18, 0.43)^a^

^a^significant association; Abbreviations: BMI, body mass index; WC, waist circumference; WHtR, waist-to-height ratio.

The overall results demonstrated that in boys only in participants with a high risk score (≥90^th^ percentile) significant associations were observed between anthropometric variables with the composite cardiometabolic score. In girls, significant associations were observed in the extremes of the risk score (low risk: <10^th^ percentile, and high risk: ≥90^th^ percentile).

**Fig 3:**
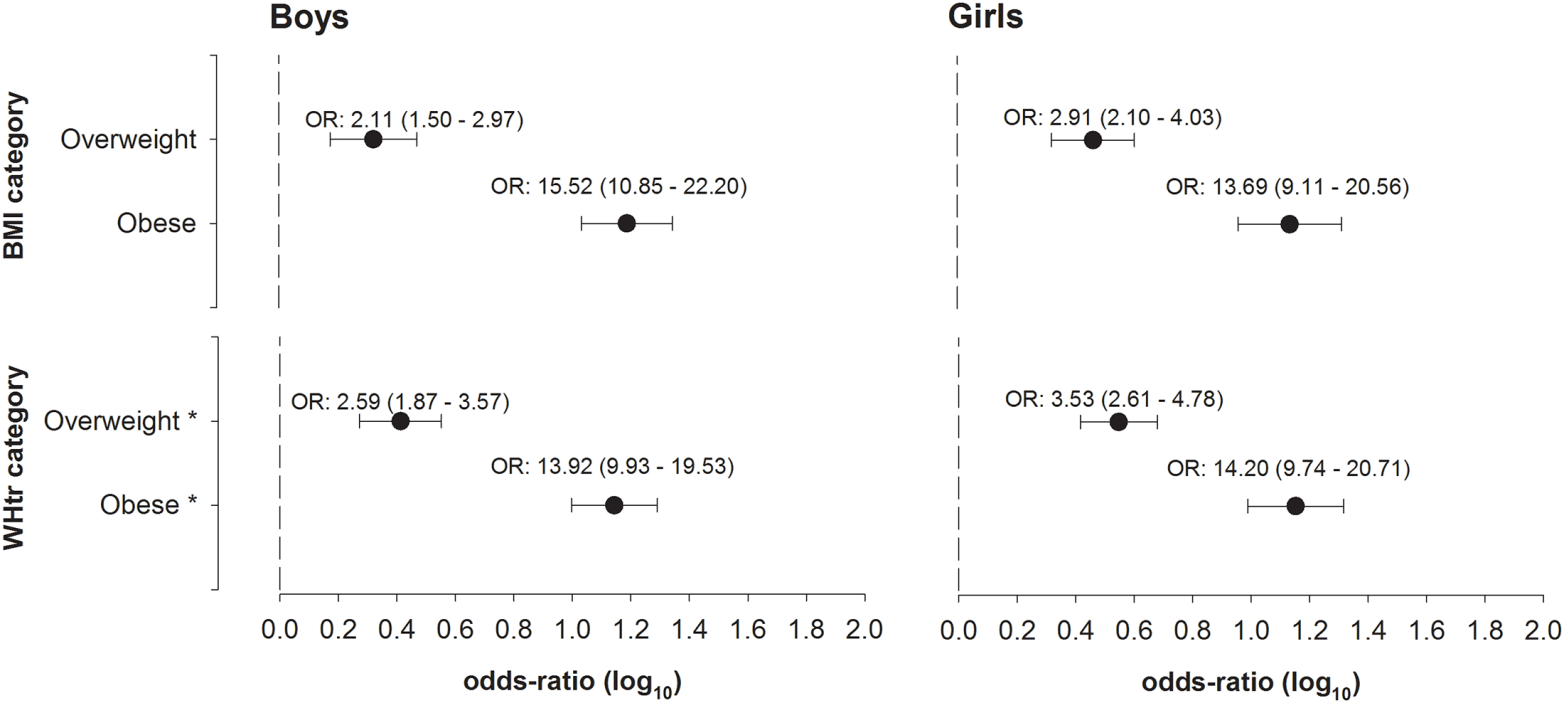
Odds-ratio for increased cardiometabolic composite z-score (>1SD) according to body mass index and waist-to-height ratio for boys (n = 2064) and girls (n = 2191). *WHtR categories were defined to categorize the same proportion of children/adolescents as the BMI categories [overweight, boys (P69): 0.45; girls (P72): 0.46; Obese, boys (P87): 0.50, girls (P91): 0.52].

Fig 3 shows the odds-ratios for participants being categorized at increased clustered metabolic risk (composite z-score>1SD) stratified by BMI and WHtR.

Overall 18.5 of boys and 19.2% of girls were overweight while 12.8 of boys and 9.0% of girls were obese, according to the WHO criteria. Using the IOTF criteria [30] we verified that 17.0% and 16.5% of boys and girls, respectively, were overweight and that 8.1% of boys and 7.1% of girls were obese. Being overweight was associated with more than twofold increased odds for having clustered metabolic risk in both sexes whereas being obese was associated with a more than about 14 times increased risk compared with normal-weight participants.

We defined WHtR categories that allowed classifying the same proportion of children and adolescents with overweight and obesity, as being classified as overweight and obese by BMI. For overweight, the cutoff corresponded to 0.45 in boys and 0.46 in girls and was associated with significantly increased odds of about 2.6 and 3.5, for cardiometabolic risk in boys and girls, respectively. The WHtR cutoffs that corresponded to the BMI category of obesity were 0.50 for boys and 0.52 for girls. These individuals had about 14 times higher odds of being categorized as having clustered metabolic risk compared with those of normal-weight.

Stepwise logistic regression analyses were performed to identify exposure variables associated with increased cardiometabolic risk score. Variables included in analysis were age, BMI/WC/WHtR, and BMI^2^/WC^2^/WHtR^2^. Table 5 show the final models and ROC analyses that were further conducted to verify each model discriminative capability.

**Table 5:** Logistic regression model and respective discriminative capability for the physical independence predictors by age category and sex

	Boys			Girls		
	*BMI*	*WC*	*WHtR*	*BMI*	*WC*	*WHtR*
**Logistic Model**						
(a) constant	-4.473	-5.245	-5.016	-6.723	-8.604	-6.325
(β_1_) age	-0.178	-0.247		-0.210	-0.219	-0.061
(β_2_) BMI / WC / WHtR*	0.241	0.094		0.483	0.187	11.556
(β_3_) BMI^2^ / WC^2^ / WHtR^2^*			15.721	-0.006	-0.001	
**Discriminative capability**						
AUC / (SD)	0.738 (0.017)	0.732 (0.017)	0.719 (0.017)	0.729 (0.017)	0.715 (0.017)	0.700 (0.017)
Probability criterion	0.20691	0.16789	0.18583	0.15571	0.17904	0.15506
Sensitivity (%)	53.3	59.4	51.8	60.8	53.2	55.1
Specificity (%)	86.4	80.5	85.2	76.6	82.3	77.5

Abbreviations: BMI, body mass index; WC, waist circumference; WHtR, waist-to-height ratio; AUC, area under the receiver-operating characteristics curve

Y = a + β_1_ * age (years) + β_2_ * BMI / WC / WHtR + β_3_ * BMI^2^ / WC^2^ / WHtR^2^

Probability = *e*^y^ / (1+ *e*^y^)

For all models the areas under the curve (AUC) were higher than 0.7 and no significant differences were found between sexes and anthropometric variables for the AUC.

The sensitivity of the models ranged from 51.8% (boys, WHtR) to 60.8% (girls, BMI). The corresponding specificity values ranged from 76.6% (girls, BMI) to 86.4% (boys, BMI).

Table 6 shows the optimal cutoffs criteria stratified by age and sex, derived from the logistic regression equations presented in table 5.

**Table 6:** Sex and age^a^-specific optimal criterion for body mass index, waist circumference, and waist-to-height ratio derived from logistic regression equations.

	Body Mass Index (kg/m^2^)	Waist Circumference (cm)	Waist-to-Height Ratio
**Boys**			
8 years	18.9	60.1	0.47
9 years	19.7	62.7	0.47
10 years	20.4	65.4	0.47
11 years	21.2	68.0	0.47
12 years	21.9	70.6	0.47
13 years	22.6	73.3	0.47
14 years	23.4	75.9	0.47
15 years	24.1	78.5	0.47
16 years	24.9	81.2	0.47
17 years	25.6	83.8	0.47
**Girls**			
8 years	17.4	60.7	0.44
9 years	18.2	62.9	0.45
10 years	18.9	65.1	0.45
11 years	19.7	67.4	0.46
12 years	20.5	69.7	0.46
13 years	21.4	72.2	0.47
14 years	22.3	74.7	0.47
15 years	23.2	77.4	0.48
16 years	24.2	80.2	0.48
17 years	25.2	83.1	0.49

^a^ Each age was categorized by the midpoint of an age. For example, the group of children with 8 years old included all the children between 7.50 years and 8.49.

It was additionally investigated whether combining different anthropometric variables provided a better discriminative capability. Stepwise logistic regressions including age, BMI, WC, and WHtR as the dependent variables were conducted by sex. In both boys and girls age (boys: β = -0.227, p<0.001; girls: β = -0.196, p<0.001), BMI (boys: β = 0.107, p = 0.007; girls: β = 0.004, p = 0.004), and WC (boys: β = 0.056, p<0.001; girls: β = 0.037, p = 0.011) were retained in the model. For the boys the AUC was 0.739 (0.017) and for girls it was 0.725 (0.017).

## Discussion

The results from the present study suggest a similar and low precision of BMI, WC, and WHtR in classifying young people as having increased clustered cardiometabolic risk. We also found that the magnitude of the association between anthropometric variables and cardiometabolic risk was stronger in overweight and obese children and adolescents compared to their normal-weight peers. In this manuscript it was demonstrated that being overweight was associated with more than twofold increased odds for having clustered cardiometabolic risk in both boys and girls while being obese was associated with a more than 14 times increased risk compared with their normal-weight peers.

Previous investigations have suggested that both BMI and WC or WHtR perform similarly when predicting a cluster of cardiometabolic risk factors [9,17,18,31]. Our results extend these observations, suggesting that the magnitude of associations between WC, WHtR and BMI with clustered metabolic risk is similar for all anthropometric variables. They also perform similar in their ability to classify those with increased clustered cardiometabolic risk. In agreement with others [14,17,32] we also observed strong associations between BMI with WC and WHtR, partly explaining the similarity in the magnitudes of associations.

We also observed that the associations between BMI, WC, and WHtR with cardiometabolic risk factors were non-linear, suggesting that at lower levels of adiposity (lower BMI/WC/WHtR) the magnitude of associations between surrogate measures and risk factors are weak as indicated by the shallow slope whereas a greater magnitude of association with a steeper slope is evident at higher BMI, WC, or WHtR. This observation is supported for a variety of cardiovascular risk factors which appear to remain steady in normal BMI/adiposity ranges and only increases at the higher end of the BMI/adiposity distribution [32]. We therefore stratified our sample according to the WHO BMI category (normal versus overweight and obese) and examined the magnitudes of associations in the normal-weight and overweight/obese groups, respectively. In these analyses, a markedly steeper positive slope for the associations between anthropometric variables and all cardiometabolic risk factors was observed in children and adolescents that were overweight or obese. Taken together this suggests that, although BMI, WC, and WHtR may not be good predictors of CVD risk factors, at higher levels of adiposity the cluster of cardiovascular risk factors increases with increasing BMI, WC, or WHtR. This was further emphasized by the increased odds for being classified as having increased cardiometabolic risk (>1SD) in those that were overweight or obese and in participants with increased and very increased WHtR compared with normal weight and normal WHtR participants, respectively. Interestingly, we additionally observed that the association between anthropometric variables with the composite cardiometabolic score was higher and significant in participants with high cardiometabolic risk (≥90^th^ percentile).

The results from the ROC analysis indicated that the AUC did not differ between anthropometric variables nor between sexes. This suggests that BMI, WC, WHtR perform similar in diagnosing children and adolescents at increased cardiometabolic risk. However, the accuracy for using these anthropometric variables when classifying participants at increased cardiometabolic risk was far from excellent as indicated by the AUC values (<0.75). Additionally it was verified that combining anthropometric variables did not represent higher AUC in both boys and girls. Regardless, the AUC are similar to other investigations [33,34] that have observed lower AUC for cardiometabolic risk variables to diagnose increased IMT (AUC: 0.60–0.66). Magnussen et. al [1] have observed an AUC of 0.65 when using pediatric values of BMI to diagnose adult metabolic syndrome. Further, the sensitivity of the models that included anthropometric variables was low suggesting that these anthropometric variables perform poorly for correct classification of young people with increased cardiometabolic risk. It was recently suggested that BMI and WC could be used to identify the clustering of risk factors in pediatric ages, although, similar to our findings the best cutoffs had a low sensitivity.[18] Likewise, Freedman et al.[10] observed that existing BMI cutoffs had a high specificity but a low sensitivity when used as screening tool for individual cardiovascular risk factors. Together with previous findings our results suggest that anthropometric variables may correctly identify young people without increased cardiometabolic risk (i.e. true negatives), whereas these variables, appears to misclassify a large proportion of children and adolescents with increased cardiometabolic risk. These observations may be clinically important and suggest that direct measurements of the risk factors are needed rather than relying on anthropometric proxies when identifying children and adolescents at increased cardiometabolic risk.

### Strengths and limitations

The present study used a large international database including children and adolescents aged 8–17 years old. Previous studies have been conducted in smaller convenient samples from a specific region. Geographical region may be important when determine the prevalence of clustered metabolic risk in pediatric ages [35].

The main limitation of the present investigation is the use of different protocols when measuring for WC. One of the studies (NHANES) included measured WC using the NIH [23] protocol while the others used the WHO approach [22]. To solve this limitation we used a conversion equation [24] so that all WC analyses were conducted with the same WC procedure. However, while conversion equations may be biased it is unlikely this will affect the main conclusions from our study. Finally, as our study is cross-sectional we cannot infer causality of our results.

In summary, the magnitudes of associations between BMI, WC, and WHtR with clustered cardiometabolic risk are similar and differ according to obesity status, with stronger associations being observed in overweight and obese participants comparing to their normal weight peers. The associations between BMI, WC, and WHtR with the composite risk score were higher in boys than in girls. Combining different anthropometric variables does not translate into a better precision in classifying children and adolescents at increased risk. Anthropometric variables used as proxies for cardiometabolic risk will misclassify a large proportion of children and adolescents with increased cardiometabolic risk. However, these anthropometric variables may be used as a noninvasive approach that may be useful as a prescreening tool, and direct measurements of the risk factors of interest are needed for screening and when used as a diagnostic tool.
